# Supplementary material for: Differential requirements of androgen receptor in luminal progenitors during prostate regeneration and tumor initiation
Source: eLife. 2018 Jan 15;7:e28768. doi: 10.7554/eLife.28768 (PMC5807048; doi:10.7554/eLife.28768)
Supplement: Figure 1—source data 1. [file elife-28768-fig1-data1.docx]

**Figure 1 source data. Quantitation of CARNs and AR-deleted CARNs *in vivo*.**

**A. Flow-sorting analysis of CARNs and AR-deleted CARNs**

| *Nkx3.1^CreERT2/+^; R26R-YFP/+* | | *Nkx3.1^CreERT2/+^; Ar^flox/Y^; R26R-YFP/+* | |
| --- | --- | --- | --- |
| Mouse ID | Percentage | Mouse ID | Percentage |
| 2709 | 0.22% | 2701 | 0.38% |
| 2710 | 0.20% | 2718 | 0.27% |
| 2711 | 0.35% | 8701 | 0.34% |
| 8706 | 0.62% | 8702 | 0.24% |
| 8708 | 0.42% | 8703 | 0.30% |
| Mean ± SD | 0.36 ± 0.17% | Mean ± SD | 0.31 ± 0.06% |

| **B. Marker analysis of CARNs and AR-deleted CARNs** | | | | | |  |
| --- | --- | --- | --- | --- | --- | --- |
| **AR** | | | | | |  |
| Genotype | Mouse ID | | Total YFP^+^ cells | AR^+^YFP^+^ cells (%) | | |
| *Nkx3.1^CreERT2/+^; R26R-YFP/+* | 2725, 9902, 9905 | | 223 | 223 (100%) | | |
| *Nkx3.1^CreERT2/+^; Ar^flox/Y^; R26R-YFP/+* | 2729, 2730, 9901, 9903 | | 395 | 51 (12.9%) | | |
|  | | | | | | |
| **CK8** | | | | | | |
| Genotype | Mouse ID | | Total YFP^+^ cells | CK8^+^YFP^+^ cells (%) | | |
| *Nkx3.1^CreERT2/+^; R26R-YFP/+* | 2724, 2725, 2726, 8082 | | 113 | 113 (100%) | | |
| *Nkx3.1^CreERT2/+^; Ar^flox/Y^; R26R-YFP/+* | 2728, 2729, 2730 | | 281 | 281 (100%) | | |
|  | | | | | | |
| **CK18** | |  | |  |  |  |
| Genotype | Mouse ID | | Total YFP^+^ cells | CK18^+^YFP^+^ cells (%) | | |
| *Nkx3.1^CreERT2/+^; R26R-YFP/+* | 9902, 9905, 2725, 8082 | | 127 | 127 (100%) | | |
| *Nkx3.1^CreERT2/+^; Ar^flox/Y^; R26R-YFP/+* | 9901, 9903, 9904, 2730 | | 148 | 148 (100%) | | |
|  | | | | | | |
| **CK5** | |  | |  |  |  |
| Genotype | Mouse ID | | Total YFP^+^ cells | CK5^+^YFP^+^ cells (%) | | |
| *Nkx3.1^CreERT2/+^; R26R-YFP/+* | 2724, 2725, 2726, 8082 | | 108 | 0 (0%) | | |
| *Nkx3.1^CreERT2/+^; Ar^flox/Y^; R26R-YFP/+* | 2728, 2729, 2730 | | 222 | 0 (0%) | | |
|  | | | | | | |
| **p63** |  | | | | | |
| Genotype | Mouse ID | | Total YFP^+^ cells | p63^+^YFP^+^ cells (%) | | |
| *Nkx3.1^CreERT2/+^; R26R-YFP/+* | 9902, 9905, 2724 | | 107 | 0 (0%) | | |
| *Nkx3.1^CreERT2/+^; Ar^flox/Y^; R26R-YFP/+* | 9901, 9903, 9904, 2730 | | 170 | 0 (0%) | | |
