# Supplementary material for: Differential requirements of androgen receptor in luminal progenitors during prostate regeneration and tumor initiation
Source: eLife. 2018 Jan 15;7:e28768. doi: 10.7554/eLife.28768 (PMC5807048; doi:10.7554/eLife.28768)
Supplement: Figure 2—source data 1. [file elife-28768-fig2-data1.docx]

**Figure 2 source data. Quantitation of YFP^+^ cells during regeneration.**

| **A. Analysis of YFP^+^ cell clusters during regeneration** | | | | | | | | |
| --- | --- | --- | --- | --- | --- | --- | --- | --- |
| **0 days regeneration (regressed prostate)** | | | | | | | | |
| Genotype | Mouse ID | YFP^+^ cells | | | YFP^+^ cell clusters (% ± SD) | | | |
|  |  | Total | AR^+^ | AR^–^ | 1 cell | 2 cell | 3 or 4 cells | > 4 cells |
| *Nkx3.1^CreERT2/+^; R26R-YFP/+* | 2725, 9902, 9905 | 223 | 223 | 0 | 137  (66.1 ± 12.9%) | 56  (22.7 ± 6.3%) | 30  (11.0 ± 7.1%) | 0 |
| *Nkx3.1^CreERT2/+^; Ar^flox/Y^; R26R-YFP/+* | 2729, 2730, 9901, 9903 | 395 | 51 |  | 45 | 6 | 0 | 0 |
|  |  |  |  | 344 | 252  (71.9 ± 5.6%) | 68  (20.3 ± 3.2%) | 24  (7.8 ± 4.3%) | 0 |
|  | | | | | | | | |
| **4 days regeneration** | | | | | | | | |
| Genotype | Mouse ID | YFP^+^ cells | | | YFP^+^ cell clusters (% ± SD) | | | |
|  |  | Total | AR^+^ | AR^–^ | 1 cell | 2 cell | 3 or 4 cells | > 4 cells |
| *Nkx3.1^CreERT2/+^; R26R-YFP/+* | 1437, 2706, 2707 | 136 | 136 | 0 | 67  (50.0 ± 5.1%) | 36  (28.1 ± 7.1%) | 33  (21.9 ± 11.8%) | 0 |
| *Nkx3.1^CreERT2/+^; Ar^flox/Y^; R26R-YFP/+* | 1447, 1448, 2716 | 145 | 23 |  | 15 | 8 | 0 | 0 |
|  |  |  |  | 122 | 81  (61.6 ± 16.6%) | 38  (36.2 ± 18.1%) | 3  (2.2 ± 3.8%) | 0 |
|  | | | | | | | | |
| **7 days regeneration** | | | | | | | | |
| Genotype | Mouse ID | YFP^+^ cells | | | YFP^+^ cell clusters (% ± SD) | | | |
|  |  | Total | AR^+^ | AR^–^ | 1 cell | 2 cell | 3 or 4 cells | > 4 cells |
| *Nkx3.1^CreERT2/+^; R26R-YFP/+* | 1439, 1440, 1441 | 238 | 238 | 0 | 117  (51.9 ± 13.8%) | 72  (28.9 ± 6.9%) | 44  (17.7 ± 11.6%) | 5  (1.5 ± 2.6%) |
| *Nkx3.1^CreERT2/+^; Ar^flox/Y^; R26R-YFP/+* | 1443, 1445, 1446 | 152 | 28 |  | 15 | 7 | 6 | 0 |
|  |  |  |  | 124 | 67  (54.0 ± 7.8%) | 40  (32.3 ± 5.8%) | 17  (13.7 ± 3.8%) | 0 |
|  | | | | | | | | |

| **14 days regeneration** | | | | | | | | |
| --- | --- | --- | --- | --- | --- | --- | --- | --- |
| Genotype | Mouse ID | YFP^+^ cells | | | YFP^+^ cell clusters (% ± SD) | | | |
|  |  | Total | AR^+^ | AR^–^ | 1 cell | 2 cell | 3 or 4 cells | > 4 cells |
| *Nkx3.1^CreERT2/+^; R26R-YFP/+* | 8091, 8244, 8245 | 150 | 150 | 0 | 35  (23.7 ± 10.2%) | 44  (31.0 ± 10.6%) | 61  (40.1 ± 11.4%) | 10  (5.2 ± 9.0%) |
| *Nkx3.1^CreERT2/+^; Ar^flox/Y^; R26R-YFP/+* | 8086, 8246, 8606 | 410 | 254 |  | 50 | 40 | 67 | 97 |
|  |  |  |  | 156 | 103  (66.8 ± 6.8%) | 36  (22.1 ± 5.9%) | 17  (11.1 ± 2.9%) | 0 |
|  | | | | | | | | |
| **28 days regeneration** | | | | | | | | |
| Genotype | Mouse ID | YFP^+^ cells | | | YFP^+^ cell clusters (% ± SD) | | | |
|  |  | Total | AR^+^ | AR^–^ | 1 cell | 2 cell | 3 or 4 cells | > 4 cells |
| *Nkx3.1^CreERT2/+^; R26R-YFP/+* | 9752, 9753, 9759 | 716 | 716 | 0 | 82  (13.2 ± 4.2%) | 116  (19.2 ± 5.4%) | 178  (25.1 ± 4.1%) | 340  (42.5 ± 9.4%) |
| *Nkx3.1^CreERT2/+^; Ar^flox/Y^; R26R-YFP/+* | 9750, 9754, 9756 | 264 | 156 |  | 31 | 36 | 23 | 66 |
|  |  |  |  | 108 | 75  (70.4 ± 4.5%) | 26  (23.0 ± 15.3%) | 7  (6.7 ± 11.5%) | 0 |

| **B. Marker analysis of YFP^+^ cells after regeneration** | | | | | | |
| --- | --- | --- | --- | --- | --- | --- |
| **CK5** | | | | | | |
| Genotype | Mouse ID | YFP^+^ cells (%) | | | | |
|  |  | Total | AR^+^ | AR^–^ | AR^+^CK5^+^ | AR^–^CK5^+^ |
| *Nkx3.1^CreERT2/+^; R26R-YFP/+* | 9752, 9753, 9759 | 334 | 334 | 0 | 7  (2.1%) | 0 |
| *Nkx3.1^CreERT2/+^; Ar^flox/Y^; R26R-YFP/+* | 9750, 9754, 9756 | 195 | 122 |  | 1 | 0 |
|  |  |  |  | 73 | 0 | 14  (19.2%) |
|  | | | | | | |
| **p63** | | | | | | |
| Genotype | Mouse ID | YFP^+^ cells (%) | | | | |
|  |  | Total | AR^+^ | AR^–^ | AR^+^p63^+^ | AR^–^p63^+^ |
| *Nkx3.1^CreERT2/+^; R26R-YFP/+* | 9752, 9753, 9759 | 403 | 403 | 0 | 14  (3.5%) | 0 |
| *Nkx3.1^CreERT2/+^; Ar^flox/Y^; R26R-YFP/+* | 9750, 9754, 9756 | 121 | 80 |  | 8 | 0 |
|  |  |  |  | 41 | - | 6  (14.6%) |
