# Supplementary material for: Differential requirements of androgen receptor in luminal progenitors during prostate regeneration and tumor initiation
Source: eLife. 2018 Jan 15;7:e28768. doi: 10.7554/eLife.28768 (PMC5807048; doi:10.7554/eLife.28768)
Supplement: Figure 3—source data 1. [file elife-28768-fig3-data1.docx]

**Figure 3 source data. Quantitation of BrdU incorporation and renal grafting data.**

**A. Analysis of BrdU incorporation during regeneration**

| **BrdU injected from day 1 through 4, analyzed at 28 days** | | | | | | |
| --- | --- | --- | --- | --- | --- | --- |
| Genotype | Mouse ID | YFP^+^ cells (% ± SD) | | | | |
|  |  | Total | AR^+^ | AR^–^ | AR^+^BrdU^+^ | AR^–^BrdU^+^ |
| *Nkx3.1^CreERT2/+^; R26R-YFP/+* | 9200, 9204,  9212 | 264 | 264 | 0 | 129  (50.9 ± 11.8%) | 0 |
| *Nkx3.1^CreERT2/+^; Ar^flox/Y^; R26R-YFP/+* | 9201,  9202,  9205 | 118 | 50 |  | 37 | 0 |
|  |  |  |  | 68 | 0 | 35  (62.9 ± 14.9%) |
|  | | | | | | |
| **BrdU injected from day 11 through 14, analyzed at 28 days** | | | | | | |
| Genotype | Mouse ID | YFP^+^ cells (% ± SD) | | | | |
|  |  | Total | AR^+^ | AR^–^ | AR^+^BrdU^+^ | AR^–^BrdU^+^ |
| *Nkx3.1^CreERT2/+^; R26R-YFP/+* | 8792,  8793,  8795 | 272 | 272 | 0 | 32  (11.1 ± 6.2%) | 0 |
| *Nkx3.1^CreERT2/+^; Ar^flox/Y^; R26R-YFP/+* | 8789,  8790,  8791 | 208 | 77 |  | 9 | 0 |
|  |  |  |  | 131 | 0 | 0 |

| **B. Renal grafts using 10 cells (CARNs or AR-deleted CARNs)** | | | |
| --- | --- | --- | --- |
| Genotype | Total | AR^+^ grafts recovered (%) | AR^–^ grafts recovered (%) |
| *Nkx3.1^CreERT2/+^; R26R-YFP/+* | 16 | 11 (68.8%) | N/A |
| *Nkx3.1^CreERT2/+^; Ar^flox/Y^; R26R-YFP/+* | 16 | 5 (31.2%) | 2 (12.5%) |
