# Supplementary material for: Differential requirements of androgen receptor in luminal progenitors during prostate regeneration and tumor initiation
Source: eLife. 2018 Jan 15;7:e28768. doi: 10.7554/eLife.28768 (PMC5807048; doi:10.7554/eLife.28768)
Supplement: Figure 6—source data 1. [file elife-28768-fig6-data1.docx]

**Figure 6 source data. Tumor phenotypes and marker quantitation.**

| **A. Histological analysis of NP-CARN and NPA-CARN prostate tissue** | | |  |
| --- | --- | --- | --- |
| Genotype | Mouse ID | Phenotype | |
| *Nkx3.1^CreERT2/+^; Pten^flox/flox^; R26R-YFP/+* | 0738 | Diffuse high-grade PIN (PIN III) | |
| *Nkx3.1^CreERT2/+^; Pten^flox/flox^; R26R-YFP/+* | 0739 | Diffuse high-grade PIN (PIN III) | |
| *Nkx3.1^CreERT2/+^; Pten^flox/flox^; R26R-YFP/+* | 0742 | Diffuse high-grade PIN (PIN III) | |
| *Nkx3.1^CreERT2/+^; Pten^flox/flox^; Ar^flox/Y^; R26R-YFP/+* | 0240 | Diffuse hyperplasia, focal atypia with apoptosis and mild inflammation | |
| *Nkx3.1^CreERT2/+^; Pten^flox/flox^; Ar^flox/Y^; R26R-YFP/+* | 0250 | Diffuse hyperplasia, focal atypia with apoptosis and mild inflammation | |
| *Nkx3.1^CreERT2/+^; Pten^flox/flox^; Ar^flox/Y^; R26R-YFP/+* | 0759 | Diffuse hyperplasia, focal atypia with apoptosis and mild inflammation | |

**B. Marker analysis of NP-CARN and NPA-CARN prostate tissue**

| **Ki67** | |  | | |  |  |  |
| --- | --- | --- | --- | --- | --- | --- | --- |
| Genotype | Mouse ID | | Total epithelial cells | Ki67^+^ cells (% ± SD) | | | |
| *Nkx3.1^CreERT2/+^; Pten^flox/flox^; R26R-YFP/+* | 0738, 0739, 0742 | | 5,199 | 480  (9.2 ± 0.26%) | | | |
| *Nkx3.1^CreERT2/+^; Pten^flox/flox^; Ar^flox/Y^; R26R-YFP/+* | 0240, 0250, 0759 | | 4,429 | 120  (2.7 ± 0.17%) | | | |

| **Cleaved caspase-3** | |  | | |  |  |  |
| --- | --- | --- | --- | --- | --- | --- | --- |
| Genotype | Mouse ID | | Total epithelial cells | CC3^+^ cells (% ± SD) | | | |
| *Nkx3.1^CreERT2/+^; Pten^flox/flox^; R26R-YFP/+* | 0738, 0739, 0742 | | 8,030 | 54  (0.69 ± 0.31%) | | | |
| *Nkx3.1^CreERT2/+^; Pten^flox/flox^; Ar^flox/Y^; R26R-YFP/+* | 0240, 0250, 0759 | | 6,999 | 180  (2.6 ± 0.07%) | | | |

| **C. Histological analysis of NPK-CARN and NPKA-CARN prostate tumors** | | |  |
| --- | --- | --- | --- |
| Genotype | Mouse ID | Phenotype | |
| *Nkx3.1^CreERT2/+^; Pten^flox/flox^; Kras^LSL-G12D/+^; R26R-YFP/+* | 0247 | Invasive adenosquamous  carcinoma | |
| *Nkx3.1^CreERT2/+^; Pten^flox/flox^; Kras^LSL-G12D/+^; R26R-YFP/+* | 0740 | Invasive adenosquamous  carcinoma | |
| *Nkx3.1^CreERT2/+^; Pten^flox/flox^; Kras^LSL-G12D/+^; R26R-YFP/+* | 0741 | Invasive papillary squamous cell carcinoma | |
| *Nkx3.1^CreERT2/+^; Pten^flox/flox^; Kras^LSL-G12D/+^; Ar^flox/Y^; R26R-YFP/+* | 0242 | Invasive papillary squamous cell carcinoma | |
| *Nkx3.1^CreERT2/+^; Pten^flox/flox^; Kras^LSL-G12D/+^; Ar^flox/Y^; R26R-YFP/+* | 0249 | Invasive papillary squamous cell carcinoma | |
| *Nkx3.1^CreERT2/+^; Pten^flox/flox^; Kras^LSL-G12D/+^; Ar^flox/Y^; R26R-YFP/+* | 0756 | Invasive papillary squamous cell carcinoma | |

**D. Marker analysis of NPK-CARN and NPKA-CARN prostate tumors**

| **Ki67** | |  | | |  |  |  |
| --- | --- | --- | --- | --- | --- | --- | --- |
| Genotype | Mouse ID | | Total epithelial cells | Ki67^+^ cells (% ± SD) | | | |
| *Nkx3.1^CreERT2/+^; Pten^flox/flox^; Kras^LSL-G12D/+^; R26R-YFP/+* | 0247, 0740, 0741 | | 7,534 | 1,454  (19.6 ± 2.4%) | | | |
| *Nkx3.1^CreERT2/+^; Pten^flox/flox^; Kras^LSL-G12D/+^; Ar^flox/Y^; R26R-YFP/+* | 0242, 0249, 0756 | | 7,862 | 1,493  (19.0 ± 1.3%) | | | |

| **Cleaved caspase-3** | |  | | |  |  |  |
| --- | --- | --- | --- | --- | --- | --- | --- |
| Genotype | Mouse ID | | Total epithelial cells | CC3^+^ cells (% ± SD) | | | |
| *Nkx3.1^CreERT2/+^; Pten^flox/flox^; Kras^LSL-G12D/+^; R26R-YFP/+* | 0247, 0740, 0741 | | 7,531 | 68  (0.93 ± 0.14%) | | | |
| *Nkx3.1^CreERT2/+^; Pten^flox/flox^; Kras^LSL-G12D/+^; Ar^flox/Y^; R26R-YFP/+* | 0242, 0249, 0756 | | 6,948 | 58  (0.84 ± 0.14%) | | | |

| **Synaptophysin** | |  | | | |  | |  |  |
| --- | --- | --- | --- | --- | --- | --- | --- | --- | --- |
| Genotype | Mouse ID | | Total epithelial cells | | Syn^+^ cells (% ± SD) | | | | |
| *Nkx3.1^CreERT2/+^; Pten^flox/flox^; Kras^LSL-G12D/+^; R26R-YFP/+* | 0247 | | | 3,029 | | | 0 | | |
|  | 0740 | | | 3,677 | | | 0 | | |
|  | 0741 | | | 3,563 | | | 0 | | |
|  | **Total:** | | | 10,269 | | | 0 | | |
| *Nkx3.1^CreERT2/+^; Pten^flox/flox^; Kras^LSL-G12D/+^; Ar^flox/Y^; R26R-YFP/+* | 0242 | | | 3,642 | | | 14 | | |
|  | 0249 | | | 3,316 | | | 41 | | |
|  | 0756 | | | 2,371 | | | 15 | | |
|  | **Total:** | | | 9,329 | | | 70  (0.75 ± 0.44%) | | |
