# Supplementary material for: Differential requirements of androgen receptor in luminal progenitors during prostate regeneration and tumor initiation
Source: eLife. 2018 Jan 15;7:e28768. doi: 10.7554/eLife.28768 (PMC5807048; doi:10.7554/eLife.28768)
Supplement: Supplementary file 1. [file elife-28768-supp1.docx]

**Supplementary file 1. Primers and antibodies used in this study.**

**A. Primers used for mouse genotyping**.

| **Allele** | **Amplicon size** | **Forward** | **Reverse** |
| --- | --- | --- | --- |
| *Nkx3.1^CreERT2^* | 500bp | 5'-CAG ATG GCG CGG CAA CAC C-3' | 5'-GCG CGG TCT GGC AGT AAA AAC-3' |
| *Nkx3.1* wild-type | 500bp | 5'-CTC CGC TAC CCT AAG CAT CC-3' | 5'-GAC ACT GTC ATA TTA CTT GGA CC-3' |
| *CreER^T2^* | 500bp | 5'-CAG ATG GCG CGG CAA CAC C-3' | 5'-GCG CGG TCT GGC AGT AAA AAC-3' |
| *R26R-YFP* | 320bp | 5'-AAA GTC GCT CTG AGT TGT TAT-3' | 5'- AAG ACC GCG AAG AGT TTG TC -3' |
| *R26R* wild-type | 600bp | 5'-AAA GTC GCT CTG AGT TGT TAT-3' | 5'-GGA GCG GGA GAA ATG GAT ATG-3' |
| *Pten^flox^* | 328bp | 5'-CAA GCA CTC TGC GAA CTG AG-3' | 5'-AAG TTT TTG AAG GCA AGA TGC-3' |
| *Pten* wild-type | 156bp | 5'-CAA GCA CTC TGC GAA CTG AG-3' | 5'-AAG TTT TTG AAG GCA AGA TGC-3' |
| *Kras^LSL-G12D^* | 550bp | 5'-AGC TAG CCA CCA TGG CTT GAG TAA GTC TGC A-3' | 5'-CCT TTA CAA GCG CAC GCA GAC TGT AGA-3' |
| *Kras* wild-type | 500bp | 5'-GTC GAC AAG CTC ATG cGG GTG -3' | 5'-CCT TTA CAA GCG CAC GCA GAC TGT AGA-3' |
| *AR^flox^* | 510bp | 5'-GTT GAT ACC TTA ACC TCT GC-3' | 5'-CTT CAG CGG CTC TTT TGA AG-3' |
| *AR* wild-type | 460bp | 5'-GTT GAT ACC TTA ACC TCT GC-3' | 5'-CTT CAG CGG CTC TTT TGA AG-3' |
| *AR^flox^* (recombined) | 270bp | 5'-GTT GAT ACC TTA ACC TCT GC-3' | 5'-CTT ACA TGT ACT GTG AGA GG-3' |
| *AR^flox^* (not recombined) | 460bp | 5'-GTT GAT ACC TTA ACC TCT GC-3' | 5'-CTT ACA TGT ACT GTG AGA GG-3' |

**B. Antibodies used in this study.**

| **Antigen** | **Supplier** | **Species** | **Dilution** | **Comments** |
| --- | --- | --- | --- | --- |
| AR | Sigma A9853 | Rabbit | 1:1000 | IF (with tyramide amplification) |
| Aurora A | Abcam ab13824 | Mouse | 1:1000 | IF (with tyramide amplification); IHC |
| BrdU | AbD Serotec MCA2060 | Rat | 1:300 | IF |
| Chromogranin A | Abcam ab15160 | Rabbit | 1:1000 | IHC |
| Cleaved caspase-3 | BD Pharmingen 559565 | Rabbit | 1:100 | IF |
| CK5 | Covance SIG-3475 | Chicken | 1:500 | IF |
| CK5 | Covance PRB-160P | Rabbit | 1:500 | IF |
| CK8 | Developmental Studies Hybridoma Bank, clone TROMA-1 | Rat | 1:100 | IF |
| CK18 | Abcam ab668, clone C-04 | Mouse | 1:100 | IF |
| Foxa1 | Abcam ab55178 | Mouse | 1:100 | IF |
| Foxa2 | Abnova H00003170-M12 | Mouse | 1:50 | IHC |
| GFP | Abcam ab13970 | Chicken | 1:1000 | IF |
| GFP | Roche 11814460001 | Mouse | 1:100 | IF |
| Ki67 | eBiosciences 14-5698, clone SolA15 | Rat | 1:100 | IF |
| Kras | Abcam ab84573 | Rabbit | 1:100 | IF (can also detect Hras and Nras) |
| p63 | Santa Cruz sc-8431 | Mouse | 1:500 | IF (discontinued by the supplier) |
| phospho-Akt | Cell Signaling 3787 | Rabbit | 1:100  1:500 | IF (1:500, with tyramide amplification) |
| PSA | Dako M0750, clone ER-PR8 | Mouse | 1:50 | IF |
| Synaptophysin | BD Transduction Laboratories 611880 | Mouse | 1:1000 | IF (with tyramide amplification); IHC |

Abbreviations: IF, immunofluorescent staining; IHC, immunohistochemical staining
