# Supplementary material for: Differential requirements of androgen receptor in luminal progenitors during prostate regeneration and tumor initiation
Source: eLife. 2018 Jan 15;7:e28768. doi: 10.7554/eLife.28768 (PMC5807048; doi:10.7554/eLife.28768)
Supplement: Transparent reporting form [file elife-28768-transrepform.docx]

***eLife’s* transparent reporting form**

We encourage authors to provide detailed information *within their submission* to facilitate the interpretation and replication of experiments. If you have any questions, please contact us: [editorial@elifesciences.org](mailto:editorial@elifesciences.org).

**Sample-size estimation**

- You should state whether an appropriate sample size was computed when the study was being designed
- You should state the statistical method of sample size computation and any required assumptions
- If no explicit power analysis was used, you should describe how you decided what sample (replicate) size (number) to use

Please outline where this information can be found within the submission (e.g., page numbers or figure legends), or explain why this information doesn’t apply to your submission:

No statistical methods were used to pre-determine sample size (page 21).

For *in vivo* analyses of CARNs, based on our previous publication (Wang *et al*., Nature 2009), we used at least 3 mice for each group (control vs experimental); we used additional animals for analyses involving regressed prostates (n=4 or 5), due to their small size. For cell of origin experiments involving the NP-CARN and NPK-CARN models, we used 3 mice per cohort, based on the similar phenotypes of the NP and NPK models as previously described (Floc’h *et al.* Cancer Res. 2012; Aytes *et al.,* PNAS 2013). These numbers are stated within the Results (pages 6-11) as well as in the source data files for Figures 1, 2, 3, and 6.

**Replicates**

- You should report how often each experiment was performed
- You should include a definition of biological versus technical replication
- The data obtained should be provided and sufficient information should be provided to indicate the number of independent biological and/or technical replicates
- If you encountered any outliers, you should describe how these were handled
- Criteria for exclusion/inclusion of data should be clearly stated
- High-throughput sequence data should be uploaded before submission, with a private link for reviewers provided (these are available from both GEO and ArrayExpress)

Please outline where this information can be found within the submission (e.g., page numbers or figure legends), or explain why this information doesn’t apply to your submission:

Each in vivo experiment was performed once with at least 3-5 biological replicates. For RNA-sequencing and bioinformatic analyses, 2 biological replicates were used for each group. For assays involving cell lines, data shown correspond to representative results from 3-5 technical replicates for each condition (APCA vs. ADCA, with and without DHT) from 1 of the 2 biological replicates. All information is presented in the source data files for Figures 1, 2. 3. 4, and 6, as well as in the Results (pages 6-11) and Figure legends (pages 32-35). RNA-sequencing data have been submitted to the Gene Expression Omnibus database under GSE99233, which can be accessed by reviewers at the private link: <https://www.ncbi.nlm.nih.gov/geo/query/acc.cgi?acc=GSE99233>, with the token "atipyiiqzxwflyd".

**Statistical reporting**

- Statistical analysis methods should be described and justified
- Raw data should be presented in figures whenever informative to do so (typically when N per group is less than 10)
- For each experiment, you should identify the statistical tests used, exact values of N, definitions of center, methods of multiple test correction, and dispersion and precision measures (e.g., mean, median, SD, SEM, confidence intervals; and, for the major substantive results, a measure of effect size (e.g., Pearson's r, Cohen's d)
- Report exact p-values wherever possible alongside the summary statistics and 95% confidence intervals. These should be reported for all key questions and not only when the p-value is less than 0.05.

Statistical analysis was performed using the Statistical Package for the Social Sciences (SPSS). Data distribution was assessed by the Kolmogorov-Smirnov test. Arcsine transformation was performed on data with non-normal distribution. Independent t-test or Fisher’s Exact Test was performed for comparison between 2 independent groups as appropriate. These methods are described under “Statistical analyses” (page 21). Raw data are shown in Supplemental Tables S1, S2, and S4. Experimental values are presented as mean +/- SD in the Results (pages 6-11) and Figure legends (pages 31-33).

Details of the statistical analyses are as follows:

1. Flow-sorting analysis of CARNs and AR-deleted CARNs – Figure 1C, n=5 biological replicates for each group; Komolgorov-Smirnov test – normal distribution (p=0.2 for both groups); Independent t-test – Levene test not significant, and therefore equal variance assumed (not significant, p=0.505).
2. Analysis of BrdU incorporation during regeneration (D1-D4 BrdU injection) – Figure 3E, BrdU positive population, n=3 biological replicates for each group; – Komolgorov-Smirnov test – non-normal distribution (p<0.0001 for both groups); Arcsine transformation was performed; Independent t-test – Levene test not significant, and therefore equal variance assumed (not significant, p=0.336).
3. Analysis of BrdU incorporation during regeneration (D11-D14 BrdU injection) – Figure 3F, BrdU positive population, n=3 biological replicates for each group – Komolgorov-Smirnov test – non-normal distribution (p<0.0001 for control; no value for AR-deleted group); Arcsine transformation was performed; Independent t-test – Levene test significant, and therefore equal variance not assumed (significant, p=0.027).
4. Comparison of successful vs unsuccessful grafts from CARNs and AR-deleted CARNs - Figure 3H, n=16 biological replicates for each group; Fisher’s Exact Test (significant, p=0.003).
5. Bioinformatic analyses – Figure 5, differential gene expression signatures were defined as a list of genes ranked by differential expression between two phenotypes of interest estimated using a 2-sample 2-tailed Welch t-test (for n ≥ 3) or fold-change (for n < 3); For comparison of a mouse gene signature with a human gene signature, mouse genes were mapped to their corresponding human orthologs based on the homoloGene database (NCBI). Signatures were compared using Gene Set Enrichment Analysis (GSEA), with the significance of enrichment estimated using 1,000 gene permutations.
6. Proliferation (Ki67) and apoptosis (CC3) indices: comparison between NP-CARN and NPA-CARN prostate tissue – Figure 6B, n=3 biological replicates for each group; Komolgorov-Smirnov test – non-normal distribution (p<0.0001 for both groups); Arcsine transformation was performed; Independent t-test – Levene test not significant, and therefore equal variance assumed (proliferation – significant, p<0.0001; apoptosis – significant, p=0.002).
7. Proliferation (Ki67) and apoptosis (CC3) indices and Synaptophysin-positive comparison between NPK-CARN and NPKA-CARN prostate tissue – Figure 6D,F, n=3 biological replicates for each group; Komolgorov-Smirnov test – non-normal distribution (p<0.0001 for both groups); Arcsine transformation was performed; Independent t-test – Levene test not significant, and therefore equal variance assumed (proliferation – not significant; apoptosis – not significant, p=0.507; synaptophysin – significant, p=0.028).

Please outline where this information can be found within the submission (e.g., page numbers or figure legends), or explain why this information doesn’t apply to your submission:

(For large datasets, or papers with a very large number of statistical tests, you may upload a single table file with tests, Ns, etc., with reference to page numbers in the manuscript.)

**Additional data files (“source data”)**

- We encourage you to upload relevant additional data files, such as numerical data that are represented as a graph in a figure, or as a summary table
- Where provided, these should be in the most useful format, and they can be uploaded as “Source data” files linked to a main figure or table
- Include model definition files including the full list of parameters used
- Include code used for data analysis (e.g., R, MatLab)
- Avoid stating that data files are “available upon request”

Please indicate the figures or tables for which source data files have been provided:

We have included source data files for Figures 1, 2, 3, 4, and 6.
